# Efficacy and safety of combination versus single-agent immunotherapy for BCG-unresponsive non-muscle-invasive bladder cancer

**DOI:** 10.3389/fimmu.2026.1896444

**Published:** 2026-07-28

**Authors:** Ying Zhou, Xu Yang, Tingting Tian, Bo Yang, Jinyang Cheng, Yanqing Liu, Dongxin Tang, Yang Liu, Yanju Li, Feiqing Wang

**Affiliations:** 1 College of First Clinical Medicine, Guizhou University of Traditional Chinese Medicine, Guiyang, Guizhou, China; 2 Clinical Medical Research Center, The First Affiliated Hospital of Guizhou University of Traditional Chinese Medicine, Guizhou, China; 3 Department of Hematology Oncology, Affiliated Hospital of Guizhou Medical University, Guiyang, Guizhou, China

**Keywords:** BCG-unresponsiveness, bladder cancer, immunotherapy, monoclonal antibodies, NMIBC

## Abstract

**Background:**

Bacillus Calmette–Guérin (BCG) is the standard adjuvant therapy for high-risk non–muscle-invasive bladder cancer (NMIBC); however, a substantial proportion of patients develop BCG-unresponsive disease with limited bladder-preserving options. The objective of this study was to determine whether combination immunotherapy provides superior clinical efficacy and safety compared with single-agent immunotherapy for patients with BCG-unresponsive NMIBC.

**Methods:**

We conducted a systematic analysis of studies retrieved from PubMed, the Cochrane Library, Web of Science, Embase, Google Scholar, and MEDLINE searched up to December 2025. Eligible studies included single-arm trials and randomized controlled trials (RCTs) enrolling patients with pathologically confirmed BCG-unresponsive NMIBC treated with single-agent or combination immunotherapy. The intervention featured eight core drugs, including immune checkpoint inhibitors (PD-1 inhibitors), adenovirus vectors, and IL-15 superagonist Nogapendekin alfa inbakicept (N-803), administered primarily every 3 weeks. Primary outcomes were complete response rate (CRR) and progression-free survival (PFS). Secondary outcomes included high-grade recurrence, bladder preservation rate (BPR), and adverse events (AEs). Pooled analysis utilized fixed or random-effects models based on I^2^ heterogeneity.

**Results:**

Ten studies (7 single-arm, 3 RCTs) involving 768 patients were included. For combination versus single-agent immunotherapy, the 3-month CRRs were 68% (95% CI: 63–75%) and 47% (95% CI: 43–51%), respectively. At 12 months, CRRs were 50% (95% CI: 43–59%) for combination and 28% (95% CI: 23–34%) for monotherapy. The 12-month PFS rate was significantly higher with combination therapy (90%; 95% CI: 85–94%) than with monotherapy (66%; 95% CI: 60–71%). Combination therapy achieved a BPR of 92.5% (95% CI: 87–98%). Regarding safety, any AEs occurred in 29.6% (95% CI: 14–46%) of the combination group compared with 87.4% (95% CI: 85–90%) in the monotherapy group. Limitations include the use of single-arm trial data, which lacks blinded evaluation, and moderate-to-severe heterogeneity in efficacy outcomes.

**Conclusion:**

Combination immunotherapy is associated with improved short and intermediate-term oncologic outcomes compared with single-agent immunotherapy in BCG-unresponsive NMIBC, with acceptable safety profiles. These findings support further investigation of combination strategies as bladder-preserving treatments; however, well-designed randomized trials are required before routine clinical adoption.

**Systematic Review Registration:**

https://www.crd.york.ac.uk/prospero/, identifier CRD420251269272.

## 1 Introduction

Bladder cancer (BC) is a common malignancy of the urinary system worldwide. In 2022, approximately 614, 000 new cases were reported globally, ranking ninth among all cancers; during the same period, there were an estimated 220, 000 deaths, making it the second leading cause of mortality among urological malignancies (1). In China, the incidence of BC exceeds that of other urinary system tumors, and it also ranks second among urological malignancies in Western countries (2). Non–muscle-invasive bladder cancer (NMIBC) represents the predominant subtype, accounting for approximately 75% of newly diagnosed cases, with tumors typically confined to the urothelium or lamina propria (3–5). Diagnosis primarily relies on cystoscopy, and the standard initial treatment is transurethral resection of bladder tumor (TURBT), which serves both diagnostic staging and therapeutic purposes (6). Although NMIBC generally has a more favorable prognosis than muscle-invasive bladder cancer (MIBC), it is characterized by a high risk of recurrence and progression, with approximately 10–30% of cases advancing to MIBC. In contrast, MIBC is associated with poor survival outcomes and often requires radical cystectomy or multimodal treatment approaches, including radiotherapy and chemotherapy (7–9). The clinical course and long-term prognosis of NMIBC are influenced by several key factors, including tumor grade (low vs high), pathological stage (Ta, T1, or carcinoma *in situ* [CIS]), tumor multiplicity, size, and the presence of CIS (10, 11).

According to the 2016 European Association of Urology (EAU) guidelines, patients with intermediate-risk disease are recommended to receive 1 year of full-dose intravesical Bacillus Calmette-Guérin (BCG) therapy, regardless of whether immediate postoperative instillation is administered following TURBT (12). BCG, an attenuated strain of *Mycobacterium bovis*, exerts its antitumor effects by inducing a localized immune response within the bladder mucosa, thereby facilitating the eradication of residual tumor cells and reducing recurrence risk (13). To date, BCG remains the gold standard adjuvant therapy for high-risk NMIBC, with multiple clinical trials demonstrating a reduction in recurrence rates of up to 50% compared with TURBT alone (14–16). However, despite its widespread use, BCG therapy has important limitations. Approximately 70% of patients achieve complete remission after the initial induction course (17), yet even with adequate maintenance therapy, 20–40% subsequently develop disease recurrence or progression (18). These patients are classified as having BCG-unresponsive or BCG-refractory disease, representing a particularly challenging clinical scenario with limited effective treatment options (19).

For patients who fail to respond to BCG therapy, particularly those with high-risk disease, early radical cystectomy remains the most effective curative option and may confer a survival benefit (20). However, as a complex and highly invasive surgical procedure, radical cystectomy is associated with a substantial risk of perioperative complications and mortality, and it can profoundly impair urinary function and long-term quality of life (21). Given these challenges, there is an urgent need to develop effective bladder-preserving treatment strategies for patients with BCG-unresponsive disease (22). In recent years, immunotherapy has shown promising antitumor activity in this setting (23, 24). Its principal advantage lies in harnessing the host immune system to target tumor cells, offering high specificity, a generally manageable safety profile, and the potential for durable therapeutic responses (25, 26). Despite the relatively high incidence of NMIBC and the effectiveness of current treatment strategies in many patients, a substantial proportion still exhibit suboptimal responses to standard BCG therapy. Consequently, the development of innovative and minimally invasive therapeutic approaches to address this unmet clinical need has become a central focus in urological oncology. The ultimate goal is to achieve both bladder preservation and improved oncological outcomes, while maintaining oncological safety as a fundamental priority. In this context, the present study aimed to systematically evaluate the efficacy and safety of immunotherapy in patients with BCG-unresponsive NMIBC, with the objective of informing bladder-preserving strategies and optimizing clinical decision-making.

## 2 Methods

This study was conducted in accordance with the PRISMA (Preferred Reporting Items for Systematic Reviews and Meta-Analyses) guidelines (27). The study protocol was prospectively registered in the International Prospective Register of Systematic Reviews (PROSPERO; registration number CRD420251269272).

### 2.1 Literature search strategy

#### 2.1.1 Eligibility criteria

Eligible studies included randomized controlled trials and cohort studies published in both domestic and international journals. For non-randomized studies, clear baseline characteristics and adequate group comparability were required. The study population consisted of patients with a pathological or cytological diagnosis of bladder cancer who were clearly defined as BCG non-responders, including those with disease recurrence or progression after treatment, or those who discontinued therapy due to intolerance without achieving remission. No restrictions were applied regarding age, sex, or race. Interventions included PD-1/PD-L1 inhibitors and other immunotherapeutic approaches administered as either monotherapy or combination therapy, with clearly defined treatment protocols and comparator groups. Eligible studies were required to report at least one clinically relevant efficacy outcome (e.g., objective response rate or complete response rate) or safety outcome (e.g., incidence of adverse events), with clearly defined evaluation criteria. Finally, only studies with complete extractable data were included.

#### 2.1.2 Exclusion criteria

Studies were excluded based on the following criteria. Regarding literature type, animal studies, *in vitro* experiments, case reports, reviews, and conference abstracts were excluded. For study populations, studies were excluded if the diagnosis of bladder cancer was unclear, if patients were not confirmed to be BCG non-responders, or if patients had other concurrent malignancies or severe comorbidities that could interfere with outcome assessment. With respect to interventions, studies that did not involve immunotherapy, lacked a clearly defined intervention protocol, or provided insufficient information on the therapeutic agents were excluded. In terms of outcome measures, studies were excluded if they did not report clearly defined efficacy or safety outcomes, lacked standardized evaluation criteria, or reported only laboratory-based indicators without clinical endpoints. For data quality, studies with missing key data that could not be obtained, studies containing data errors, and duplicate publications (in which case only the most complete or highest-quality report was retained) were excluded. Finally, studies were excluded if the overall methodological quality was considered insufficient or if the full text was not accessible.

### 2.2 Information sources and search strategy

A comprehensive literature search was conducted using the following keywords: “non–muscle-invasive bladder cancer” and “carcinoma *in situ*, “ combined with terms related to BCG treatment failure, including “BCG recurrence, “ “BCG intolerance, “ “BCG resistance, “ “BCG drug resistance, “ and “BCG failure.” Electronic databases, including PubMed, the Cochrane Library, Web of Science, Embase, Google Scholar, and MEDLINE, were systematically searched from their inception to December 31, 2025. The study selection and screening process was performed in strict accordance with the PRISMA guidelines (27).

### 2.3 Data extraction and quality assessment

Study screening and data extraction were performed independently by two investigators to ensure accuracy and objectivity. Any discrepancies arising during the selection or evaluation process were resolved through discussion, and when necessary, a third reviewer was consulted to reach consensus. This approach was implemented to maintain methodological rigor and minimize potential bias. The primary outcome measures were complete response rate (CRR) and progression-free survival (PFS). Secondary outcomes included high-grade recurrence-free rate, bladder preservation rate, and the incidence of adverse events. Extracted data comprised key study characteristics, including first author, year of publication, country of origin, and tumor-related features.

Among the ten included studies, seven were single-arm trials and three were randomized controlled trials. The risk of bias in non-randomized studies was assessed using the ROBINS-I tool (28), while randomized controlled trials were evaluated using the Cochrane Risk of Bias 2.0 tool (29).

### 2.4 Statistical analyses

Systematic analysis of the included studies was performed, and the results were presented as forest plots. Statistical heterogeneity among studies was assessed using the Q test and the I^2^ statistic. A *P* value <0.1 in the Q test was considered indicative of significant heterogeneity. I^2^ values of 25–50%, 50–75%, and 75–100% were interpreted as low, moderate, and high heterogeneity, respectively. A fixed-effects model was applied when heterogeneity was low (*P* > 0.1 and I^2^ <50%), whereas a random-effects model was used in the presence of significant heterogeneity (*P* ≤ 0.1 and I^2^ ≥50%). In addition, the risk of bias across included studies was systematically evaluated to ensure the robustness of the findings.

The primary analyses focused on CRR at 3, 6, and 12 months, as well as 12-month PFS and bladder preservation rate. Subgroup analyses were conducted to compare outcomes between treatment strategies. Differences between groups were assessed using the chi-square test, with a two-sided *P* value <0.05 considered statistically significant. All statistical analyses were performed using R software (version 4.5; Meta package) and Review Manager (RevMan, version 5.4).

## 3 Results

### 3.1 Search strategies and study selection

**Figure 1:**
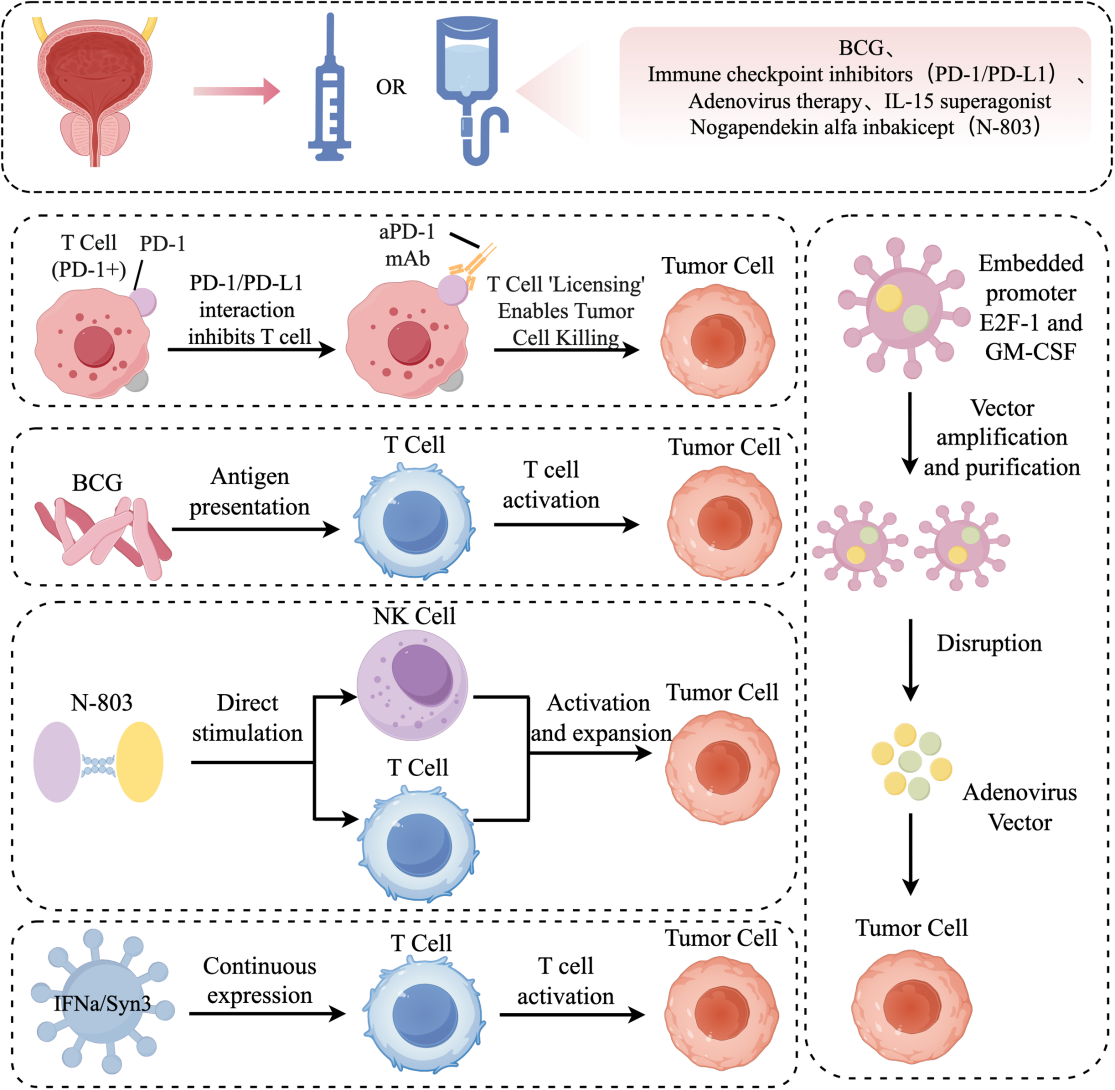
The immunotherapy mechanisms in BCG-unresponsive non–muscle-invasive bladder cancer.

The mechanistic framework of this study illustrates that immune checkpoint inhibitors (PD-1 inhibitors), BCG, the IL-15 superagonist Nogapendekin alfa inbakicept (N-803), rAd–IFNa/Syn3, and adenoviral vector–based therapies activate T cells and natural killer cells to mediate tumor cell killing through key immune pathways (**Figure 1**).

**Figure 2:**
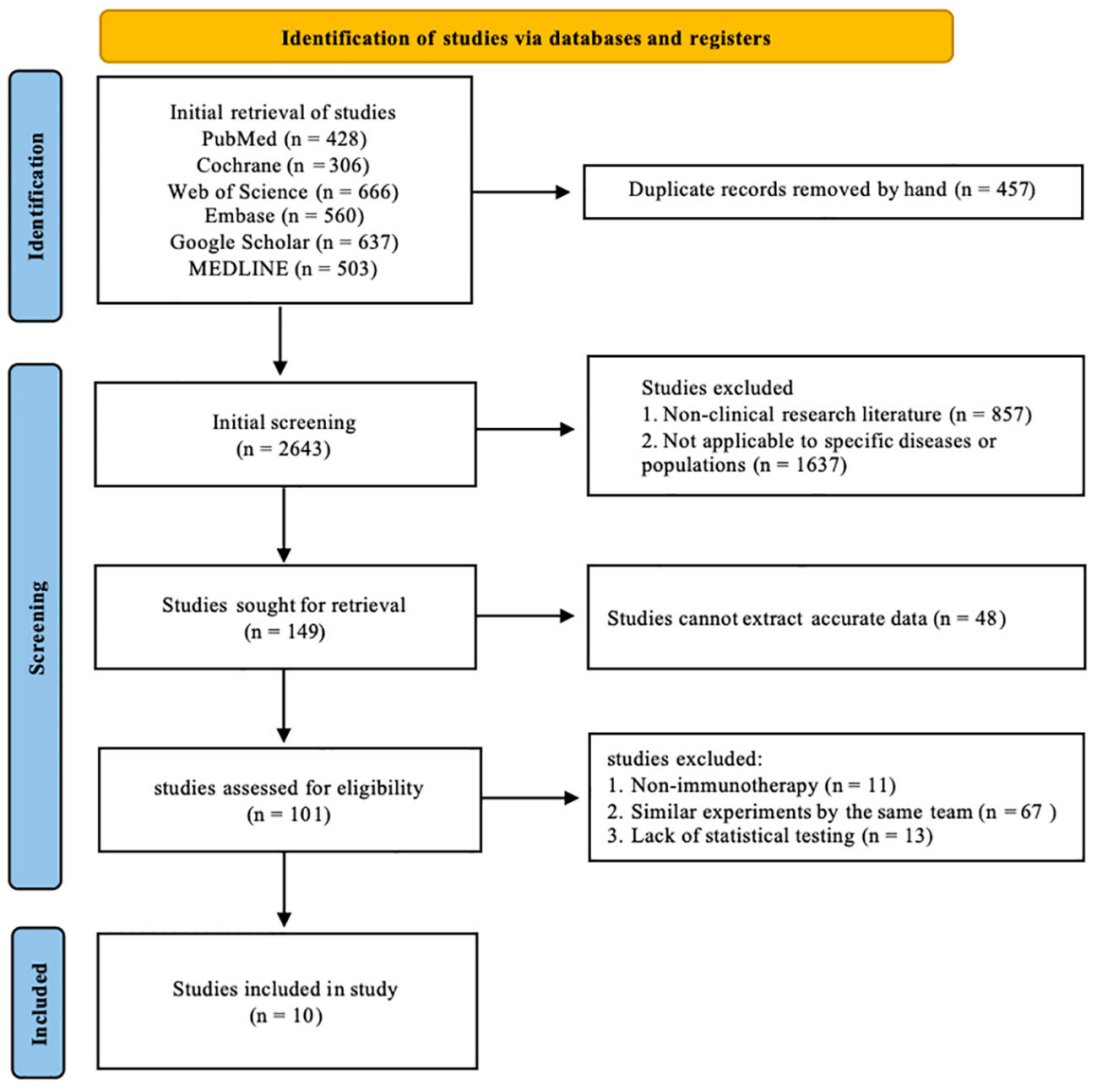
PRISMA flow diagram of the literature screening process.

A total of 3, 100 records were initially identified from PubMed, the Cochrane Library, Web of Science, Embase, Google Scholar, and MEDLINE. After removal of 457 duplicate records, 2, 643 articles remained for screening. Of these, 857 non-clinical studies and 1, 637 studies not relevant to the target population or disease were excluded based on titles and abstracts, leaving 149 articles for full-text assessment. Subsequently, 48 studies were excluded due to insufficient or non-extractable data. A total of 101 studies met the initial inclusion criteria; however, 11 were further excluded for lack of relevance to immunotherapy, 67 were excluded due to overlapping or duplicate cohorts from the same research groups, and 13 were excluded because of insufficient statistical analysis. Ultimately, 10 studies were included in the final analysis (**Figure 2**).

### 3.2 Characteristics of the studies

The 10 included NMIBC clinical studies (seven from the United States, one from Canada, one from Italy, and one from Greece) evaluated a total of eight core therapeutic agents, encompassing monoclonal antibodies, adenoviral vector–based therapies, IL-15 superagonist Nogapendekin alfa inbakicept (N-803), and combination regimens. Among these, PD-1/PD-L1 inhibitors represented the predominant class, including pembrolizumab, atezolizumab, and durvalumab. Adenoviral vector–based therapies included nadofaragene firadenovec and rAd-IFNa/Syn3. Additional agents comprised the IL-15 superagonist Nogapendekin alfa inbakicept and the monoclonal antibody–based gene therapy kretuximab.

Treatment administration was most commonly performed every 3 weeks, although some regimens were administered weekly. Treatment duration ranged from 6 weeks to 2 years. Most studies stratified patients according to baseline tumor stage (CIS, Ta, T1, or combinations thereof), with only one study not reporting baseline staging. Three combination regimens were identified, including atezolizumab plus BCG, durvalumab plus BCG, and nogapendekin alfa inbakicept plus BCG, as well as a cross-category combination of kretuximab plus pembrolizumab. Across all studies, therapeutic efficacy and safety were evaluated in different NMIBC subtypes. Detailed treatment protocols, trial registration numbers, and baseline patient characteristics are summarized in **Table 1**.

**Table 1:** Baseline characteristics and design of the 10 included studies.

Details of the authors	Registration number	Baseline stage	Treatment plan
Stephen A Boorjian 2021 America (30)	NCT02773849	CIS (n = 81), Ta (n = 35), CIS + Ta(n = 21), T1(n = 12), CIS + T1(n = 5)	75ml nadofaragene firadenovec (3 × 10^11^ viral particles per mL)
Arjun V Balar 2021 America (31)	NCT02625961	CIS(n = 64), Ta (n = 25), T1 (n = 12)	200mg Pembrolizumab, Once every three weeks, for a maximum duration of 24 months.
Peter C Black 2023 Canada (32)	NCT02844816	CIS cohort (n = 74): CIS + Ta 74%, CIS + Ta 74%, CIS + T1 72%, CIS + Ta + T1 80%; Non-CIS cohort (n = 55): Ta 55%, T1 55%, Ta + T1 60%	1200mg atezolizumab, Once every 3 weeks, with a maximum of 51 weeks.
Andrea Necchi 2024 Italy (33)	NCT02625961	Ta (n = 75), T1 (n = 57)	200mg Pembrolizumab, Once every 3 weeks, a maximum of 8 cycles can be conducted.
Noah M Hahn 2023 America (34)	NCT03317158	CIS (n = 8), Ta (n = 4), CIS + Ta(n = 1), T1(n = 1), CIS + T1(n = 2)	cohort A: 1120mg, Durvalumab, Once every three weeks, a maximum of eight cycles can be conducted. cohort B: Durvalumab + BCG
Charalampos Fragkoulis 2025 Greece (35)	NCT03759496	CIS cohort (n = 22) Non-CIS cohort (n = 7)	first stage: Maximum tolerated dose: 1000 milligrams, Durvalumab, Once every 3 weeks, with a maximum of 6 weeks. second stage: The maximum dosage of Durvalumab
Brant A Inman 2023 America (36)	NCT02792192	CIS (n = 6), Ta (n = 7), T1(n = 11)	cohort A: 1200mg, atezolizumab, Once every 3 weeks, for a total of 96 weeks or 32 times. cohort B: atezolizumab+BCG
Neal D Shor 2017 America (37)	NCT01687244	CIS (n = 21), Ta (n = 4), CIS + Ta(n = 4), T1(n = 6), CIS + T1(n = 5)	75 mL rAd–IFNa/Syn3
Karim Chamie M.D. 2022 America (38)	NCT03022825	NA	cohort A: Nogapendekin alfa inbakicept(400μg) + BCG(50 mg); Once a week, for a total of 6 weeks cohort B: Nogapendekin alfa inbakicept(400μg) +BCG(50 mg); Once a week, for a total of 6 weeks cohor tC: Nogapendekin alfa inbakicept(400μg) Once a week, for a total of 6 weeks
Roger Li 2024 America (39)	NCT04387461	CIS cohort (n = 27); CIS+Ta cohort (n = 4); CIS+T1 cohort (n = 4);	Induction period: Cletuximab gene is administered intravesically once a week for six times, followed by three weekly maintenance infusions at 3, 6, 9, 12, and 18 months. If CIS or high-grade Ta type bladder cancer persists at the 3rd month, a second induction course is conducted. Pembrolizumab: Administered every 6 weeks for approximately 2 years or until disease persists or recurs.

CIS: Carcinoma in Situ; BCG: Bacillus Calmette-Guérin.

**Figure 3:**
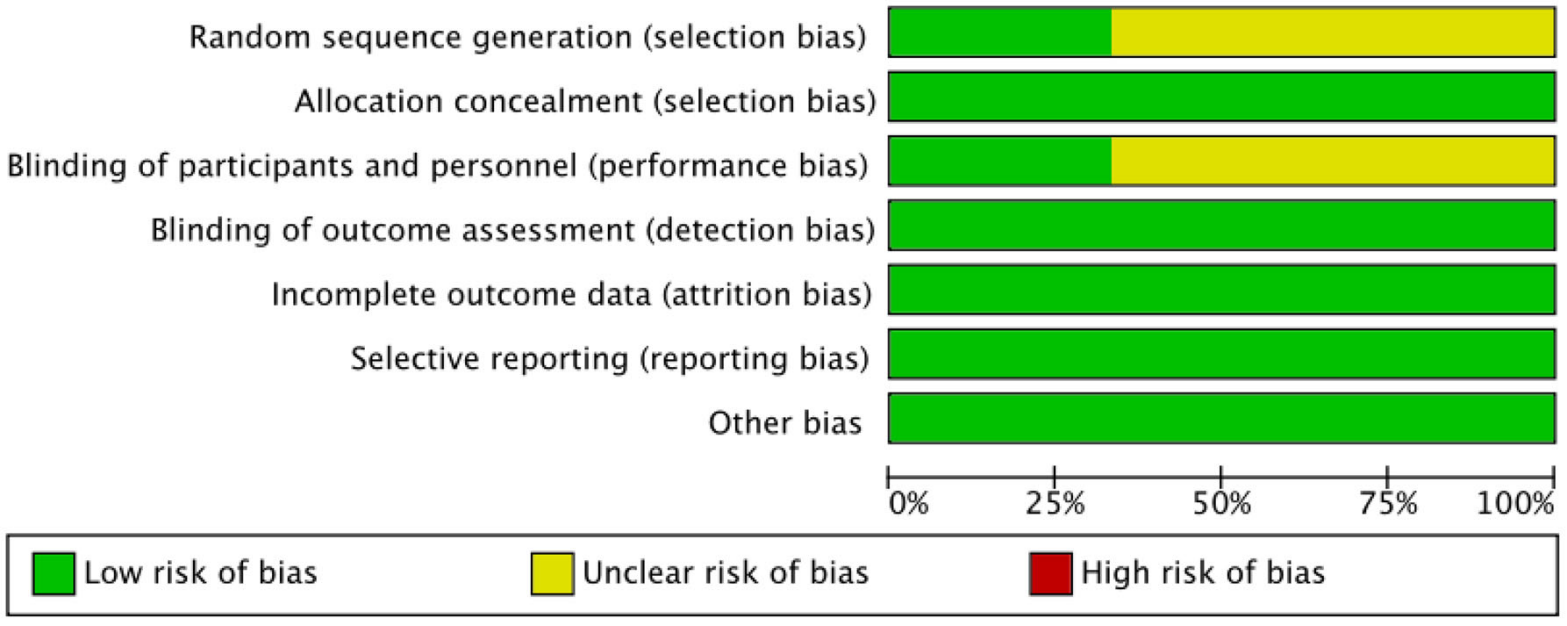
Risk assessment diagram for bias.

Overall, the analysis included seven single-arm trials and three randomized controlled trials. The risk of bias in non-randomized studies was assessed using the ROBINS-I tool (**Supplementary Table 1**), whereas randomized controlled trials were evaluated using the Cochrane Risk of Bias 2.0 tool (**Figure 3**).

### 3.3 Complete remission rate of single or combination immunotherapy

**Figure 4:**
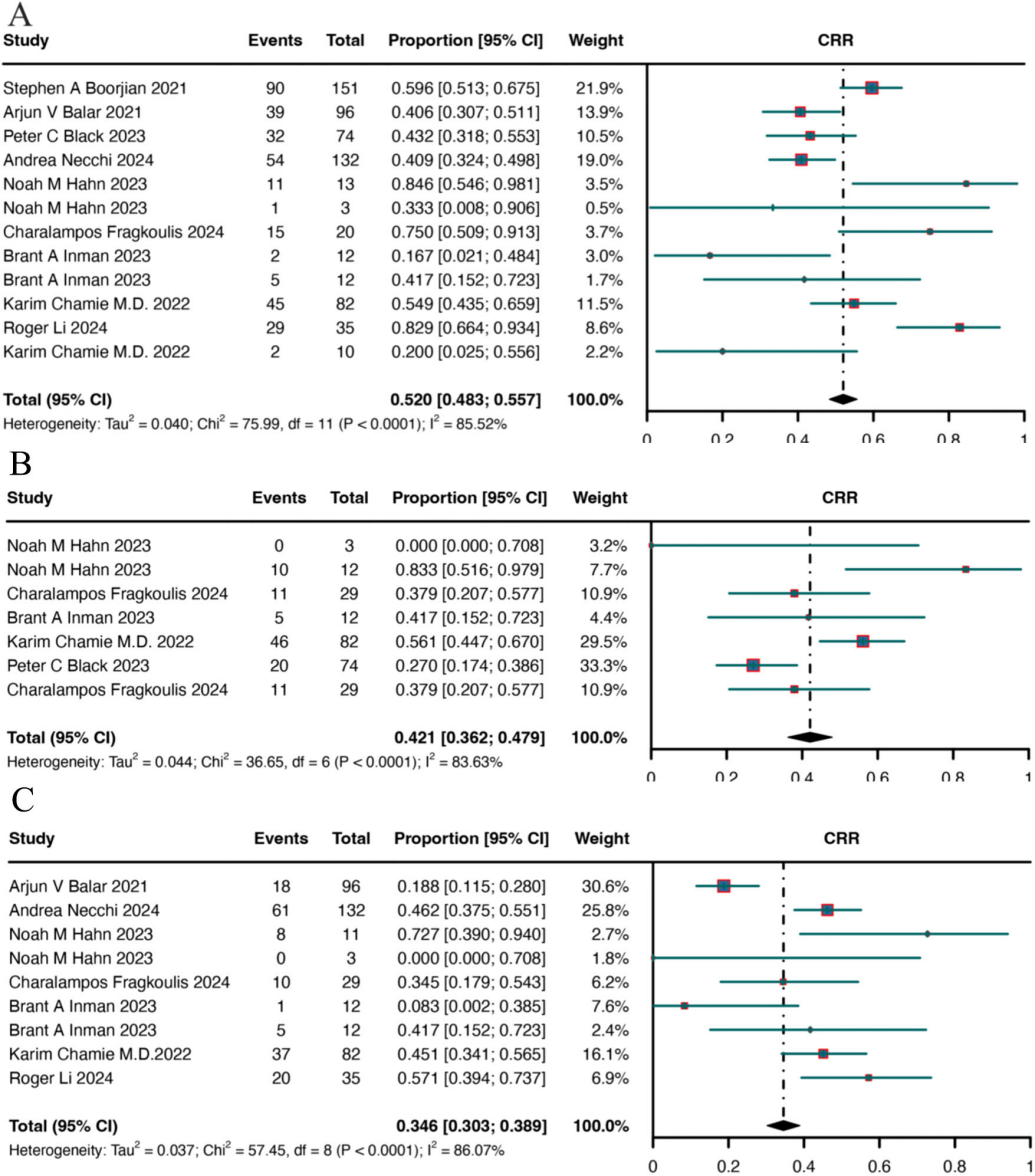
Forest plots of CRR at different time points for single versus combination immunotherapy. **(A)** 3-month CRR. **(B)** 6-month CRR. **(C)** 12-month CRR.

Across 12 trials including 640 patients, the pooled 3-month CRR was 52.0% (95% CI: 48–56%; I^2^ = 85.52%; **Figure 4A**). Subgroup analysis demonstrated a CRR of 46.7% in the single immunotherapy group (95% CI: 43–51%; I^2^ = 80.08%; **Table 2**) compared with 67.7% in the combination immunotherapy group (95% CI: 63–75%; I^2^ = 85.67%; **Table 2**), with a significantly higher response rate observed in the combination group (*P* < 0.01).

**Table 2:** Meta-regression analysis of CRR for different treatment strategies at 3, 6, and 12 months.

Treatment	3-month CRR		6-month CRR		12-month CRR	
	OR (95% CI)	*p* value	OR (95% CI)	*p* value	OR (95% CI)	*p* value
Single treatment	0.47 (0.43-0.51)	< 0.0001	0.30 (0.22-0.37)	= 0.1603	0.28 (0.23-0.34)	< 0.0001
Combined treatment	0.68 (0.60-0.75)	= 0.0006	0.60 (0.51-0.69)	= 0.0323	0.54 (0.43-0.59)	= 0.1940

CRR = complete response rate; OR = odds ratio; CI = confidence interval.

In 7 trials involving 241 patients, the pooled 6-month CRR was 42.1% (95% CI: 36–48%; I^2^ = 83.63%; **Figure 4B**). Subgroup analysis showed that the CRR was 29.6% in the single immunotherapy group (95% CI: 22–37%; I^2^ = 41.89%; **Table 2**) and 59.6% in the combination immunotherapy group (95% CI: 51–69%; I^2^ = 70.89%; **Table 2**), again demonstrating a significantly higher response rate with combination therapy (*P* < 0.01).

In 9 trials including 412 patients, the pooled 12-month CRR was 34.6% (95% CI: 30–39%; I^2^ = 86.07%; **Figure 4C**). Subgroup analysis indicated that the CRR was 28.4% in the single immunotherapy group (95% CI: 23–34%; I^2^ = 87.68%; **Table 2**) compared with 50.4% in the combination immunotherapy group (95% CI: 43–59%; I^2^ = 36.35%; **Table 2**), with the combination group showing a significantly improved response rate (*P* < 0.01).

### 3.4 Twelve-month progression-free survival of single versus combination immunotherapy

**Figure 5:**
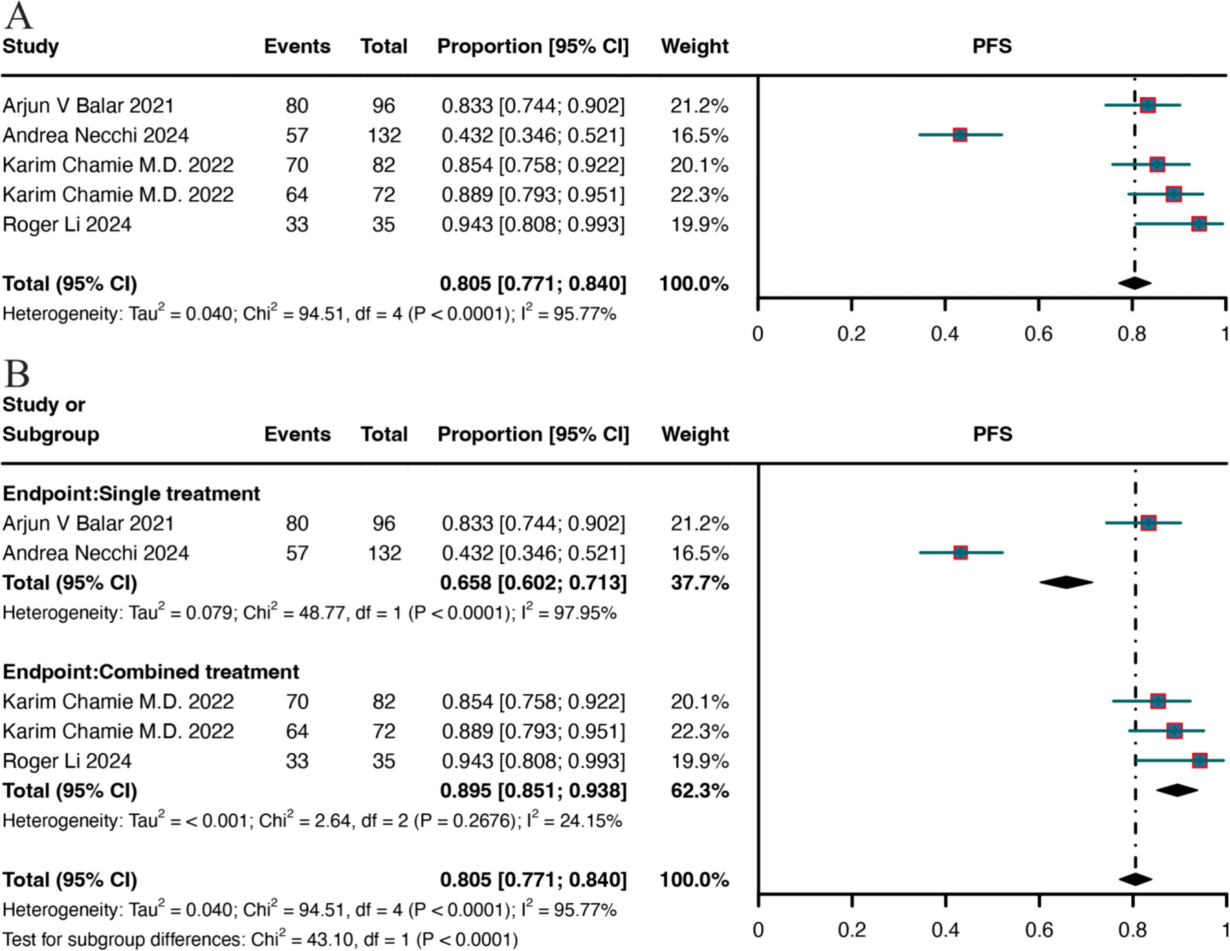
Forest plots of 12-month PFS for single versus combination immunotherapy. **(A)** Overall analysis including all trials. **(B)** Subgroup analysis comparing single and combination immunotherapy.

Across five trials including 417 patients, the pooled 12-month PFS rate was 80.5% (95% CI: 77–84%; I^2^ = 95.77%; **Figure 5A**). Subgroup analysis demonstrated that the 12-month PFS rate was 65.8% in the single immunotherapy group (95% CI: 60.2–71%; I^2^ = 97.95%; **Figure 5A**) and 89.5% in the combination immunotherapy group (95% CI: 85–94%; I^2^ = 24.15%; **Figure 5B**). The difference between groups was statistically significant, with combination therapy associated with a higher PFS rate (*P* < 0.01).

### 3.5 Bladder preservation and safety outcomes of single versus combination immunotherapy

**Figure 6:**
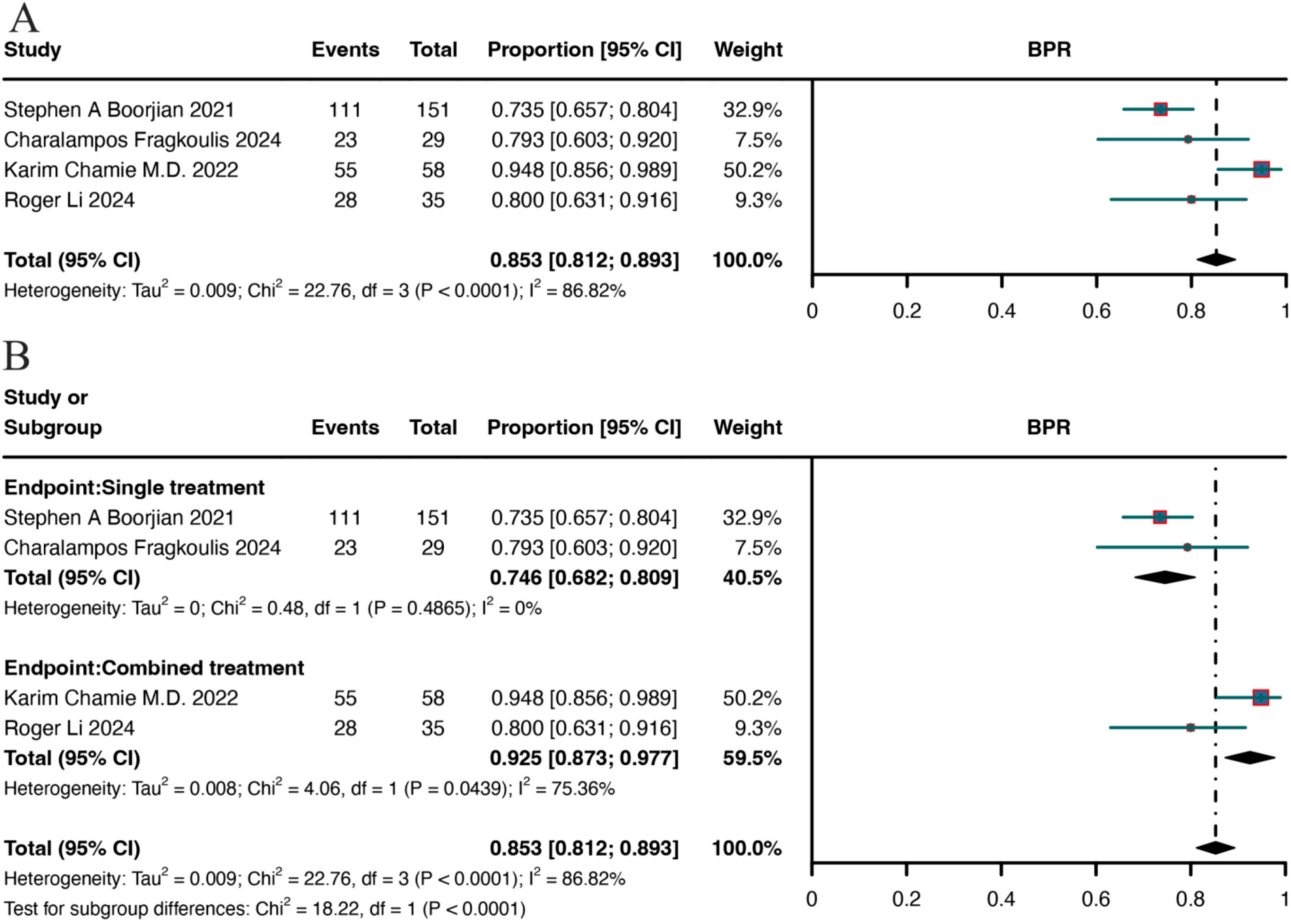
Forest plots of the pooled bladder preservation rate. **(A)** Overall analysis including all trials. **(B)** Subgroup analysis comparing single and combination immunotherapy.

Across four trials including 273 patients, the pooled bladder preservation rate (BPR) was 85.3% (95% CI: 81–90%; I^2^ = 86.82%; **Figure 6A**). Subgroup analysis demonstrated that the 12-month BPR was 74.6% in the single immunotherapy group (95% CI: 68–81%; I^2^ = 0%; **Figure 6A**) and 92.5% in the combination immunotherapy group (95% CI: 87–98%; I^2^ = 75.36%; **Figure 6B**), with a significantly higher rate observed in the combination group (*P* < 0.01).

**Figure 7:**
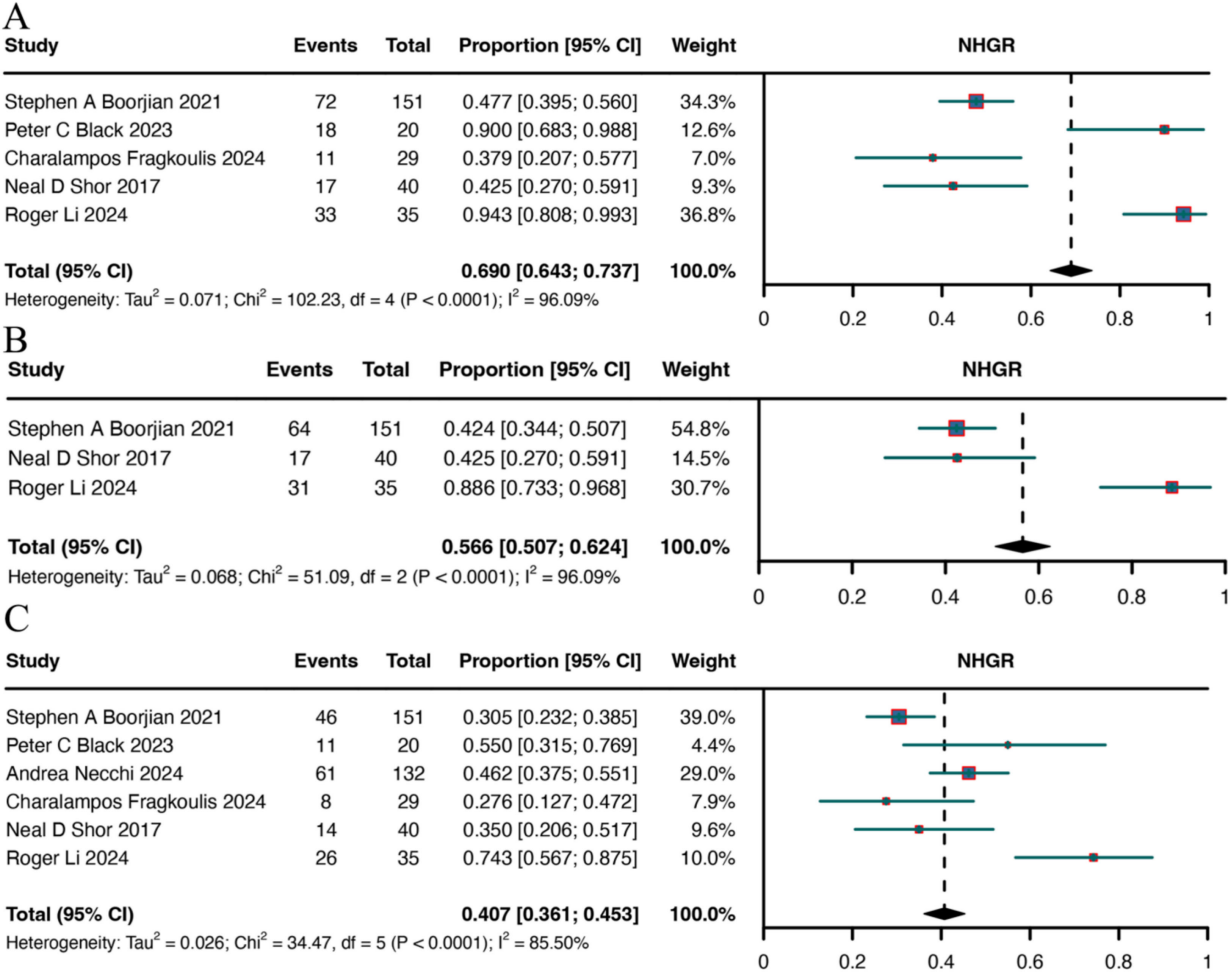
Forest plot without high-level recurrence rate. **(A)** No high-level recurrence rate within 6 months. **(B)** No high-level recurrence rate within 9 months. **(C)** No high-level recurrence rate within 12 months.

Statistically significant differences were observed at 6, 9, and 12 months (*P* < 0.0001). In addition, the incidence of non–high-grade recurrence decreased over time (**Figure 7**). A total of 671 patients from four trials were included in the safety analysis. The pooled incidence of AEs was 86.1% (95% CI: 84–89%; I^2^ = 93.49%; **Table 3**). Subgroup analysis showed that the incidence of AEs was 87.4% in the single immunotherapy group (95% CI: 85–90%; I^2^ = 91.89%; **Table 3**) compared with 29.6% in the combination immunotherapy group (95% CI: 14–46%; I^2^ = 83.59%; **Table 3**), indicating a lower overall AE rate in the combination group. Further analyses of treatment-related adverse events (TRAEs) were performed based on the safety data reported in the included studies (**Supplementary Table 2**).

**Table 3:** Summary of safety outcomes (AEs) in patients.

Project	Hererogeneity				MD (95% CI)	Sample size (Exp/Con)
	TaU_2_	CHi_2_	I_2_(%)	P-value		
Any adverse events						
Single treatment	0.039	98.69	91.89	<0.0001	0.874 (0.850-0.897)	514/646
Combined treatment	0.077	6.09	83.59	0.0136	0.296 (0.135-0.456)	9/25
Overall	0.061	153.50	93.49	<0.0001	0.861 (0.837-0.885)	523/671
Grade 3–4 adverse reactions						
Single treatment	0	3.3	0	0.6546	0.155 (0.126-0.184)	94/594
Combined treatment	0	0.36	0	0.5459	0.211 (0.151-0.272)	37/174
Overall	<0.001	6.39	0	0.0986	0.165 (0.139-0.191)	131/768

## 4 Discussion

This study comprehensively evaluated the efficacy and safety of immunotherapy in patients with NMIBC unresponsive to BCG. The findings demonstrate that combination immunotherapy consistently outperforms single-agent approaches across multiple efficacy endpoints while maintaining an acceptable safety profile. In terms of short-term efficacy, the 3-month CRR with combination therapy reached 67.7%, exceeding that of single-agent therapy (46.7%) by more than 20 percentage points, with this advantage becoming increasingly pronounced over longer follow-up durations.

BCG remains the gold standard for adjuvant therapy in high-risk NMIBC (40, 41). Although initial complete remission is achieved in approximately 70% of patients, more than half will experience recurrence or progression to a BCG-unresponsive state within one year (42, 43). These patients historically face a therapeutic dilemma: while radical cystectomy may improve survival, it is associated with perioperative complication rates exceeding 30% and can lead to substantial and irreversible impairment of urinary function, thereby significantly reducing quality of life (44, 45). In contrast, the 12-month CRR of single-agent immunotherapy is limited to approximately 28%, which is often insufficient to achieve durable tumor control. The present study demonstrates that combination immunotherapy can effectively overcome these limitations. Specifically, CRR improved to 67.7% at 3 months, 59.6% at 6 months, and 50.4% at 12 months, representing increases of 21.0, 30.6, and 22.0 percentage points, respectively, compared with single-agent therapy. Similarly, the 12-month PFS rate reached 89.5%, reflecting a 23.7-percentage-point improvement, while the bladder preservation rate increased to 92.5%, representing a 17.9-percentage-point gain. Collectively, these findings support combination immunotherapy as an effective bladder-preserving strategy for patients with BCG-unresponsive disease. This approach may be particularly valuable for elderly patients or those with significant comorbidities who are unsuitable for, or decline, radical cystectomy, as it offers a favorable balance between oncologic efficacy and quality of life, thereby expanding the therapeutic options available in clinical practice.

The results of this study further support the clinical feasibility of combination immunotherapy. Traditionally, combination strategies have been considered to carry an increased risk of additive toxicity (46). However, our findings challenge this assumption. The incidence of AEs in the combination immunotherapy group was 29.6%, markedly lower than the 84.7% observed in the single-agent group. In addition, the incidence of common TRAEs, including fatigue (20.2%), rash (10.8%), and thyroid dysfunction (9.6%), was not increased with combination therapy. This favorable safety profile may be explained by underlying mechanistic factors. Combination regimens, such as BCG plus PD-L1 inhibitors, can more precisely target the bladder tumor microenvironment through localized immune activation, thereby reducing systemic drug exposure (41, 47, 48). However, the relatively favorable safety profile observed in several combined treatment regimens may not merely be attributed to the dosing regimen; rather, it may reflect factors such as local bladder administration, differences in systemic drug exposure, patient selection criteria, and the specific treatment regimens studied. These findings challenge the conventional view that improved efficacy must come at the expense of increased toxicity, thereby addressing a major barrier to the broader clinical adoption of combination immunotherapy.

In addition, this study provides empirical evidence supporting the synergistic effects of immunotherapy in NMIBC. BCG exerts antitumor activity primarily by activating local immune responses within the bladder mucosa (49). Combination regimens, including PD-1/PD-L1 inhibitors plus BCG or adenoviral vector combined with PD-1 inhibitors, may enhance this effect through complementary mechanisms. Specifically, immune checkpoint inhibitors can reverse T-cell exhaustion and promote the infiltration of effector T cells into the tumor microenvironment, while BCG or adenoviral vector facilitate the release of tumor-associated antigens, thereby activating antigen-presenting cells and amplifying immune responses. This coordinated process establishes a positive feedback loop of antigen release, immune activation, and tumor cell killing (50–52). The sustained improvement in 12-month CRR observed with combination therapy (50.4% vs 28.4% with single-agent therapy) further supports the durability of this synergistic effect and provide clinical support for the development of combination immunotherapy strategies.

From a clinical perspective, this study not only highlights the advantages of combination immunotherapy but also provides valuable insights for individualized treatment through subgroup analyses. The 10 included studies encompassed eight core therapeutic agents and three combination regimens. The results demonstrated that dual immunotherapy achieved a 12-month PFS rate of 89.5% and the highest bladder preservation rate (92.5%), suggesting that it may represent a preferred strategy for patients with CIS or T1 disease. Furthermore, the combined application of adenoviral vector–based therapies and PD-1 inhibitors has shown a significantly higher complete response rate (82.9% at 3 months) in relapsed patients. Based on adenoviral vector therapy, the early clinical response rate has demonstrated encouraging results, but longer-term indicators such as 12-month progression-free survival rate and bladder preservation rate are more clinically significant for comparing treatment strategies in different studies.

This study provides important evidence to support the optimization of treatment strategies for high-risk NMIBC. However, several methodological limitations should be acknowledged. First, there was considerable heterogeneity among the included studies, particularly with respect to patient selection criteria, intervention protocols, and outcome assessment methods, which may have influenced the consistency of the pooled estimates. Nevertheless, this study attempted to mitigate these effects through standardized data extraction procedures and appropriate heterogeneity analyses. Second, a substantial proportion of the included studies were single-arm trials that lacked randomization and blinding, thereby increasing the potential for selection and measurement bias. Although strict inclusion and exclusion criteria and objective outcome measures were applied to enhance reliability, these inherent design limitations cannot be fully eliminated. Finally, the included data were derived from multiple independent clinical centers, with generally consistent treatment outcomes observed across studies. This consistency supports the reproducibility and clinical relevance of the findings, although further high-quality randomized controlled trials are warranted to confirm these results.

## 5 Conclusion

This study demonstrates that, in patients with high-risk NMIBC unresponsive to BCG therapy, combination immunotherapy is associated with improved short- and intermediate-term efficacy, high bladder preservation rates, and an acceptable safety profile. These findings provide important evidence to inform the development of optimized, bladder-preserving treatment strategies for this challenging patient population. Combination immunotherapy may represent a particularly valuable alternative for patients who are elderly, have significant comorbidities, or are unsuitable for or unwilling to undergo radical cystectomy. Nevertheless, further well-designed randomized controlled trials are required to validate these findings, assess long-term outcomes, and define the optimal role of combination immunotherapy in individualized treatment strategies.

## Data Availability

The original contributions presented in the study are included in the article/**Supplementary Material**. Further inquiries can be directed to the corresponding authors.
