# Supplementary material for: Efficacy and safety of combination versus single-agent immunotherapy for BCG-unresponsive non-muscle-invasive bladder cancer
Source: Front Immunol. 2026 Jul 28;17:1896444. doi: 10.3389/fimmu.2026.1896444 (PMC13457208; doi:10.3389/fimmu.2026.1896444)
Supplement: Supplementary file 1 [file SupplementaryFile1.docx]

Supplement Table 1. ROBINS-I assessment.

| Name | B1 | B2 | B3 | B4 | B5 | B6 | B7 | Overall |
| --- | --- | --- | --- | --- | --- | --- | --- | --- |
| Stephen A Boorjian 2021 | Low | Low | Low | Low | Low | Low | Low | Low |
| Arjun V Balar 2021 | Low | Low | Low | Low | Low | Low | Low | Low |
| Peter C Black 2023 | Low | Low | Low | Low | Low | Low | Low | Low |
| Andrea Necchi 2024 | Low | Low | Low | Low | Low | Low | Low | Low |
| Charalampos Fragkoulis 2025 | Low | Low | Low | Low | Low | Low | Low | Low |
| Neal D Shor 2017 | Low | Low | Low | Low | Low | Low | Low | Low |
| Roger Li 2024 | Low | Low | Low | Low | Low | Low | Low | Low |

Note: B1: Bias due to confounding; B2: Bias due to selection of participants; B3:Bias due to classification of interventions; B4:Bias due to deviations from intended interventions; B5: Bias due to missing data; B6: Bias in measurement of outcomes; B7:Bias in selection of the reported result.
