# Supplementary material for: Efficacy and safety of combination versus single-agent immunotherapy for BCG-unresponsive non-muscle-invasive bladder cancer
Source: Front Immunol. 2026 Jul 28;17:1896444. doi: 10.3389/fimmu.2026.1896444 (PMC13457208; doi:10.3389/fimmu.2026.1896444)
Supplement: Supplementary file 2 [file SupplementaryFile2.docx]

Supplement Table 2. Safety can assess the summary of AEs in patients.

| Project | Hererogeneity | | | | Mean Difference | Sample Size  (Exp/Con) |
| --- | --- | --- | --- | --- | --- | --- |
|  | TaU^2^ | Chi^2^ | I^2^(%) | *P*-value |  |  |
| Any adverse events | | | | | | |
| Single treatment | 0.039 | 98.69 | 91.89 | <0.0001 | 0.874(0.850-0.897) | 514/646 |
| Combined treatment | 0.077 | 6.09 | 83.59 | 0.0136 | 0.296(0.135-0.456) | 9/25 |
| Overall | 0.061 | 153.50 | 93.49 | <0.0001 | 0.861(0.837-0.885) | 523/671 |
| Grade 3-4 adverse reactions | | | | | | |
| Single treatment | 0 | 3.3 | 0 | 0.6546 | 0.155(0.126-0.184) | 94/594 |
| Combined treatment | 0 | 0.36 | 0 | 0.5459 | 0.211(0.151-0.272) | 37/174 |
| Overall | <0.001 | 6.39 | 0 | 0.0986 | 0.165(0.139-0,191) | 131/768 |
