# Supplementary material for: Efficacy and safety of combination versus single-agent immunotherapy for BCG-unresponsive non-muscle-invasive bladder cancer
Source: Front Immunol. 2026 Jul 28;17:1896444. doi: 10.3389/fimmu.2026.1896444 (PMC13457208; doi:10.3389/fimmu.2026.1896444)
Supplement: Supplementary file 3 [file SupplementaryFile3.docx]

Supplement Table 3. TRAE in patients for safety evaluation.

| Project | Heterogeneity | | | | Mean DIfferen  95% | Sample size |
| --- | --- | --- | --- | --- | --- | --- |
|  | Tau^2^ | Ch^i2^ | I^2^(%) | *P*-value |  | (Exp/Con) |
| Fatigue | 0.017 | 61.58 | 88.63 | <0.0001 | 0.202(0.172-0.231) | 159/647 |
| Aspartate aminotransferase increased | 0.004 | 12.43 | 75.87 | 0.0060 | 0.070(0.041-0.098) | 31/314 |
| Alanine aminotransferase increased | 0.003 | 16.35 | 75.54 | 0.0026 | 0.041(0.022-0.060) | 45/415 |
| Rash-Rash maculopapular | 0 | 0.68 | 0 | 0.9541 | 0.108(0.078-0.138) | 20/190 |
| Anaemia | 0 | 0.17 | 0 | 0.9197 | 0.105(0.061-0.148) | 56/399 |
| Pruritus | 0.002 | 5.77 | 65.33 | 0.0559 | 0.130(0.098-0.163) | 28/399 |
| Nausea | <0.001 | 5.99 | 66.59 | 0.0501 | 0.057(0.035-0.080) | 46/450 |
| Hypothyroidism | <0.001 | 3.79 | 0 | 0.5796 | 0.096(0.069-0.123) | 27/399 |
| Hypertthyroidism | 0 | 1.10 | 0 | 0.5770 | 0.065(0.041-0.089) | 30/399 |
| Dermatitis | 0 | 1.46 | 0 | 0.4820 | 0.072(0.047-0.097) | 25/399 |
| Arthralgia | 0 | 0.56 | 0 | 0.7547 | 0.061(0.038-0.085) | 61/450 |
| Diarrhoea | 0.002 | 12.12 | 58.75 | 0.0332 | 0.117(0.087-0.146) | 11/148 |
| Cough | 0 | 0.21 | 0 | 0.9017 | 0.075(0.032-0.117) | 6/249 |
| Constipation | 0 | 0.60 | 0 | 0.8969 | 0.022(0.004-0.041) | 5/249 |
| Influenza - like illness | 0 | 0.73 | 0 | 0.8652 | 0.018(0.001-0.034) | 5/249 |
| Pyrexia | 0.001 | 11.94 | 58.13 | 0.0356 | 0.026(0.011-0.041) | 21/418 |
| Hyperglycaemia | 0 | 1.39 | 0 | 0.7085 | 0.017(0.000-0.038) | 4/160 |
| Micturition urgency | 0.011 | 11.11 | 64.01 | 0.0253 | 0.172(0.126-0.218) | 48/248 |
| Urinary retention | 0.015 | 13.67 | 78.05 | 0.0034 | 0.162(0.115;0.209) | 43/219 |
| Haematuria | 0.006 | 16.35 | 69.42 | 0.0059 | 0.037(0.012-0.063) | 20/204 |
